# PET imaging of HER2 expression with an ^18^F-fluoride labeled aptamer

**DOI:** 10.1371/journal.pone.0211047

**Published:** 2019-01-25

**Authors:** Hyun Jeong Kim, Jun Young Park, Tae Sup Lee, In Ho Song, Ye Lim Cho, Ju Ri Chae, Hyungu Kang, Jong Hoon Lim, Jung Hwan Lee, Won Jun Kang

**Affiliations:** 1 Department of Nuclear Medicine, Yonsei University College of Medicine, Gangnam Severance Hospital, Seoul, Korea; 2 Department of Nuclear Medicine, Yonsei University College of Medicine, Severance Hospital, Seoul, Korea; 3 Molecular Imaging Research Center, Research Institute of Radiological and Medical Sciences, Korea Institute of Radiological and Medical Sciences, Seoul, Korea; 4 R&D Strategic Planning, Bundang CHA Medical Center, Gyeonggi-do, Korea; 5 INTEROligo Corporation, Imi-ro, Ulwang-si, Gyeonggi-do, Korea; Kyungpook National University School of Medicine, REPUBLIC OF KOREA

## Abstract

**Background/Purpose:**

Aptamers are oligonucleotide or peptide molecules that bind to a target molecule with high affinity and specificity. The present study aimed to evaluate the target specificity and applicability for *in vivo* molecular imaging of an aptamer labeled with a radioisotope.

**Methods:**

The human epidermal growth factor receptor 2 (HER2/ErbB2) aptamer was radiolabeled with ^18^F-fluoride. HER2-positive tumor cell uptake of the aptamer was evaluated in comparison to negative controls by flow cytometry and confocal microscopy. Using ^18^F-labeled HER2-specific aptamer positron emission tomography (PET), *in vivo* molecular images of BT474 tumor-bearing mice were taken at 60, 90 and 120 minutes after injection.

**Results:**

In flow cytometric analysis, HER2 aptamer showed strong binding to HER2-positive BT474 cells, while binding to HER2-negative MDA-MB231 cells was quite low. Likewise, in confocal microscopic images, the aptamer was bound to HER2-positive breast cancer cells, with minimal binding to HER2-negative cells. *In vivo* PET molecular imaging of BT474 tumor-bearing mice revealed significant higher uptake of the ^18^F-labeled HER2 specific aptamer into the tumor compared to the that of HER2-negative cell tumor(p = 0.033). HER2 aptamer was able to preferentially bind to HER2-positive breast cancer cells both *in vitro* and *in vivo*, by recognizing HER2 structure on the surface of these cells.

**Conclusion:**

The ^18^F-labeled aptamer enabled appropriate visualization of HER2 expression by human breast cancer cells. The results suggest that a radiolabeled HER2 aptamer could potentially be applied in the development of treatment strategies or in targeted therapy against HER2-positive breast cancer cells.

## Introduction

Aptamers, from the Latin “aptus,” meaning to fit, and the Greek “meros,” meaning region, are single-stranded oligonucleotides ranging from 20–90 base pairs in length. Usually derived using Systematic Evolution of Ligands by Exponential Enrichment (SELEX) methodologies [1, 2], aptamers bind to a target molecule with high affinity and specificity [3, 4]. Hence, they are regarded as ideal reagents for detecting and measuring expression of their target molecules. Aptamers have several advantages over antibodies, including reduced production costs, ease of synthesis, low toxicity, low immunogenicity, and the fact that they do not require an organism for their production [5]. Accordingly, aptamers are relatively new reagents in the field of theragnosis. Numerous aptamers have been generated against a variety of targets, such as thrombin [6], nucleolin [7], prostate-specific membrane antigen (PSMA) [8], tenascin-C (TNC) [9], and viral proteins [10]. In the therapeutic arena, Pegaptanib, a targeted anti-VEGF (vascular endothelial growth factor) aptamer [11], was approved by the FDA for treatment of macular degeneration. At present, many aptamers are undergoing preclinical and clinical phase evaluation [12], and more trials with diagnostic and therapeutic oligonucleotides are being carried out. Consequently, there is a growing demand for feasible methods with which to evaluate and verify already developed aptamers.

HER2, a well-known oncogene, is amplified or overexpressed in approximately 15–30% of breast cancers [13, 14]. It is strongly associated with a high incidence of disease recurrence and poor prognosis in several cancers [15]. Two key signaling pathways activated by HER2 are the mitogen-activated protein kinases (MAPK) pathway, which stimulates proliferation, and the phosphatidylinositol 3 kinase–protein kinase B (PI3K–Akt) pathway, which promotes tumor cell survival [16]. Accordingly, HER2 is regarded as an important therapeutic target in applicable cancers. There are well-known therapeutic monoclonal antibodies targeting HER2, such as trastuzumab and pertuzumab, which are clinically effective. Meanwhile, several DNA/RNA aptamers targeting HER2 have previously been developed via conventional SELEX and cell SELEX [17–21]. The potential pharmacological utility of a HER2 aptamer for tumor inhibition by an endocytosis-mediated mechanism was recently reported [19].

Molecular imaging refers to noninvasive, real-time visualization of biochemical events at the cellular and molecular level within living cells, tissues, and/or intact subjects [22]. PET is a representative molecular imaging modality. It is a radionuclide molecular imaging technique that enables evaluation of biochemical changes and levels of molecular targets within a living subject. PET has excellent sensitivity and a wide range of applications in basic research and preclinical arenas, and can easily be applied in the clinical field, owing to its negligible pharmacological effects. In the field of molecular imaging, the visualization of tumor targeting by aptamers is an emerging technique. Hicke et al. previously investigated molecular imaging using aptamer. They conjugated ^99m^Tc to TTA1, an aptamer for the extracellular matrix protein tenascin-C, and obtained γ-camera images of tumors *in vivo* [23]. Subsequently, PET imaging of tenascin-C with a radiolabeled single-stranded DNA aptamer was reported by Jacobson et al [24]. To the best of our knowledge, ^18^F PET imaging of a HER2 aptamer in mouse model of breast cancer has not yet been investigated.

In this study, we conjugated the radioisotope ^18^F to a HER2-specific aptamer in order to validate its target specificity and utility for *in vivo* molecular imaging.

## Materials and methods

### Ethics statement

All the described procedures were reviewed and approved by the Animal Care Use Committee at Yonsei University (IACUC 2014–0259) and were performed in accordance with the guiding principles for the Care and Use of Laboratory Animals.

### Cell culture

The HER2-positive human breast cancer cell line BT474 was used for *in vitro* and *in vivo* experiments. As a negative control for all of the experiments, the human breast cancer cell line MDA-MB231 was used. SK-BR3 and HS578T cells were used as positive and negative controls to compare HER2 expression. All cell lines were purchased from the American Type Culture Collection (ATCC, Manassas, VA, USA). The cells were maintained in minimum essential medium supplemented with 10% fetal bovine serum and antibiotics in a humidified incubator at 37°C.

### Cell lysis and western blotting

To extract cellular proteins, cells were incubated in cell lysis buffer (Invitrogen Life Technologies, Carlsbad, CA, USA) containing protease inhibitors on ice for 30 minutes. Cell lysates were clarified by centrifugation at 14,000 rpm at 4°C for 20 min. Protein concentrations were determined via the Bradford method (Thermo Fisher Scientific, Rockford, IL, USA). For western blot analysis, 30 μg of protein extract from each sample was electrophoresed on 10% SDS-PAGE gels and transferred to nitrocellulose membranes. The membranes were probed with HER2 antibody (Abcam, Cambridge, MA, USA), and ß-actin antibody (Santa Cruz Biotechnology, Santa Cruz, CA, USA) was used as a loading control. Signals were developed using an ECL chemiluminescence substrate kit (Advansta, Menlo Park, CA, USA).

### Annealing, and conjugation of aptamer

HER2 aptamers were purchased from Aptamer Sciences Inc. (APSCI, Pohang, Republic of Korea) (#SH-1194-35[25]) and Bioneer (Bioneer, Daejeon, Republic of Korea) (2-2(t)[19]). The SH-1194-35 aptamer is a modified aptamer with a 5’ amine group, which contains napthyl nucleoside to increase affinity and serum half-life (molecular weight: 14343.07). The scrambled random sequence was created using a random number table with a length equal to the that of the SH-1194-35 aptamer.

**Fig 1:**
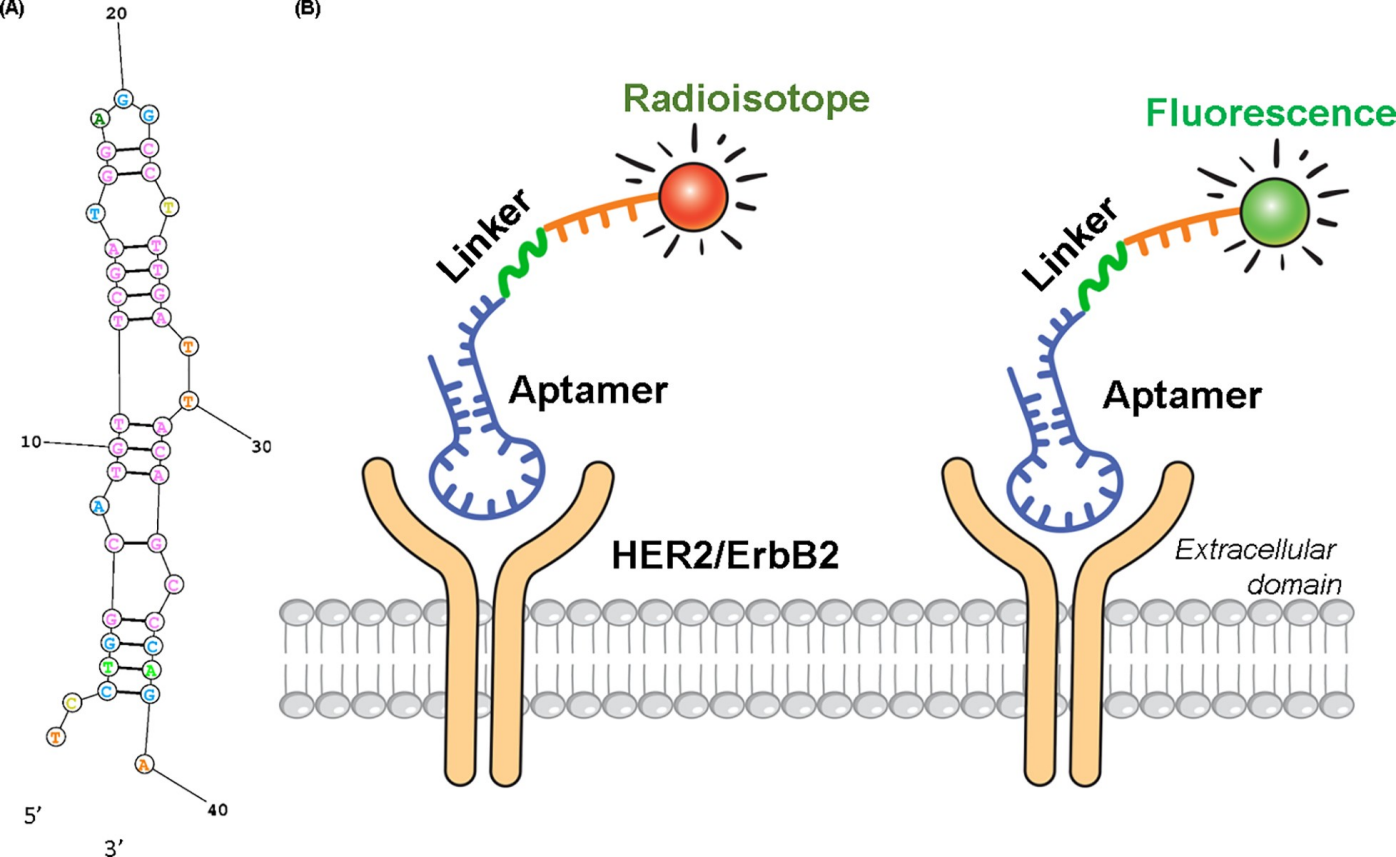
The structure of HER2 aptamer. The predicted secondary structure of aptamer SH-1194-35 based on a lowest free energy model (A) and schematic mechanism (B) of radioisotope- or fluorescence-labeled aptamer.

The aptamer was dissolved in dH2O to a final concentration of 2 μg/μL. Aptamer was heated and cooled to permit proper folding of their structures according to the manufacturer's instructions. Fluorescence-labeled single stranded DNA was purchased from Bioneer (Bioneer, Daejeon, Republic of Korea). Oligonucleotides were annealed at their calculated melting temperatures (Tm’s) and allowed to slowly cool to room temperature. The secondary folding structure and Tm of each oligonucleotide were calculated on the basis of its lowest free energy structure, using Oligocalc web servers [26, 27] (Fig 1A).

### Radiolabeling of ^18^F-Aptamer

^18^F-aptamer was synthesized according to previously reported procedures [24] with some modifications. N-succinimidyl 4-^18^F-fluorobenzoate (^18^F-SFB) was synthesized via a three-step, one-pot method [28–30] by a synthesizer (Tracerlab FXFN, GE Healthcare, Milwaukee, WI, USA). Purified ^18^F-SFB (0.78–1.48 GBq) was reconstituted in dimethylformamide. Oligonucleotides were labeled with ^18^F using click chemistry [31, 32]. Amine-terminated HER2 aptamer (5–10 nmol in PBS, pH 8.5) was added to the ^18^F-SFB residue, and the mixture was stirred for 30 minutes at 37°C. Subsequently, the mixture was purified by using reverse phase high performance liquid chromatography (HPLC) with a semi-preparative C18 column (Xbridge OST C18 10×50 mm, gradient acetonitrile/0.1M TEAA 5:95–95:5 over 20 minutes) at a flow rate of 5 mL/min, equipped with a UV (254 nm) detector and a radioactivity detector. The HPLC fraction containing ^18^F-FB aptamer was diluted with water and passed through a C18 Sep-Pak cartridge (Waters Corporation, Milford, MA). The labeled aptamer was then eluted with 500 μL of ethanol and formulated in normal saline for *in vivo* experiments. The schematic mechanismof radioisotope- or fluorescence-labeled aptamer is shown in Fig 1B.

### Confocal microscopy

BT474 or MDA-MB231 cells were seeded onto coverslips and cultured overnight. The cells, at > 80% confluence, were carefully washed and then incubated with the fluoro-labeled aptamer at a final concentration of 250 nM. After 30 minutes incubation, cells were carefully washed and mounted on slides on mounting medium containing (4’, 6-diamidino-2-phenylindole) DAPI (Vector Laboratories, Inc., Burlingame, CA, USA). The fluorescent signal was detected via LSM700 confocal microscopy (Zeiss, Jena, Germany). Excitation wavelength and emission filters were as follows: fluorescein isothiocyanate (FITC), 488 nm laser line excitation, emission BP490-555; and DAPI, 405 nm laser line excitation, emission SP490 filter.

### Flow cytometry

The specificity of the HER2 aptamer was evaluated with a fluorescence-activated cell sorting (FACS) LSR II Flow Cytometer System (BD Biosciences, San Jose, CA, USA). BT474 and MDA-MB231 cancer cells were seeded onto Petri dishes in complete medium and grown to 80% confluence. Cells were treated with trypsin, washed, and incubated at 4°C for 30 minutes with the fluoro-labeled HER2-specific aptamer or antibody (Biolegend, San Diego, CA, USA) as a control in saline containing 1% fetal bovine serum. After washing, bound aptamer molecules were detected, and cells were analyzed using a fiuorescence-activated cell sorter.

### In vivo experiment

Four-week-old female *Balb/c* nude mice were implanted subcutaneously with 17β-estradiol pellets (Innovative Research of America, Sarasota, FL, USA) at the dorsal side of the neck, with dosage release rates sufficient to permit estrogen-dependent tumorigenesis. After a few days, mice were inoculated subcutaneously with 7 × 10^6^ human breast cancer cells in the axilla. Tumors were allowed to develop for 3 weeks before imaging studies were performed. PET images were taken when the tumor was 1 cm or more. The maximal tumor size was 1.7 cm. Tumor growth was monitored by caliper measurement. Careful observations were be made a minimum of once daily for the duration of the study. General activity, body condition, hydration, interaction with cage mates and appearance were carefully observed to assess general health and well-being. During PET imaging, mouse was observed continuously for the whole imaging time. Frequency of observation was increased with the appearance of any clinical signs. The endpoints were selected to minimize the potential pain of the animal according to ethical issues. The euthanasia was performed when the tumor exceed 2 cm, the body weight decreased by more than 20%, the ulcer developed, or the condition of the animal appears to be bad. Humane euthanasia was performed using a carbon dioxide chamber.

### Ex vivo biodistribution

BT474 tumor-bearing mice were injected with ^18^F-labeled HER2 aptamer (7.4 ± 0.2 MBq) through lateral tail vein and sacrificed at 60 minutes (n = 4) after injection. Twelve tissues (blood, muscle, heart, lung, spleen, bone, liver, stomach, small intestine, large intestine, kidney, and tumor) were harvested, washed, weighed, and assayed. Blood samples were obtained by cardiac puncture, and muscle were dissected from thigh. The radioactivity of each sample was measured using a gamma counter (PerkinElmer, CA, USA). The uptake of radiotracer in tissues was expressed in counts per minute corrected with decay and normalized to the percentage injected dose per gram (%ID/g).

### ^18^F-aptamer PET imaging

We intravenously injected radiopharmaceutical-labeled aptamer into mice and performed PET imaging using a Siemens Inveon PET (Siemens Medical Solutions USA Inc., Knoxville, TN, USA). The injected dose was 13.7 ± 1.1 MBq (370 ± 30 μCi). A dynamic PET study was performed for 30 minutes with the following protocol: ten 1- minute images and four 5- minutes images. Two static studies were then performed, for 10 minutes each, at 60, 90 and 120 minutes after injection. Semi-quantification of PET signals was performed using AMIDE software (SourceForge, New York, NY, USA). The uptake of PET image was quantified based on Region of interest (ROI) analysis. 3D ROI were drawn around the edge of the tumor or organ activity by visual inspection. The mean and maximum activities were recorded from the entire ROI. Uptake analysis was performed to quantify the tracer uptake as the %ID/g in the tumors as well as in background muscle uptake. Images are presented using a false-color or gray-color scale that is proportional to tissue concentration (%ID/g) of positron-labeled probe.

### Statistical analyses

Statistical analyses were performed in SPSS version 22.0 (SPSS Inc., Chicago, Illinois). Independent sample t-test were used to analyze the variance of experimental design. Each experiment was repeated at least three times in duplicates, and statistically significant at p <0.05.

## Results

### Verification of HER2 expression and aptamer affinity for target tumor cells

**Fig 2:**
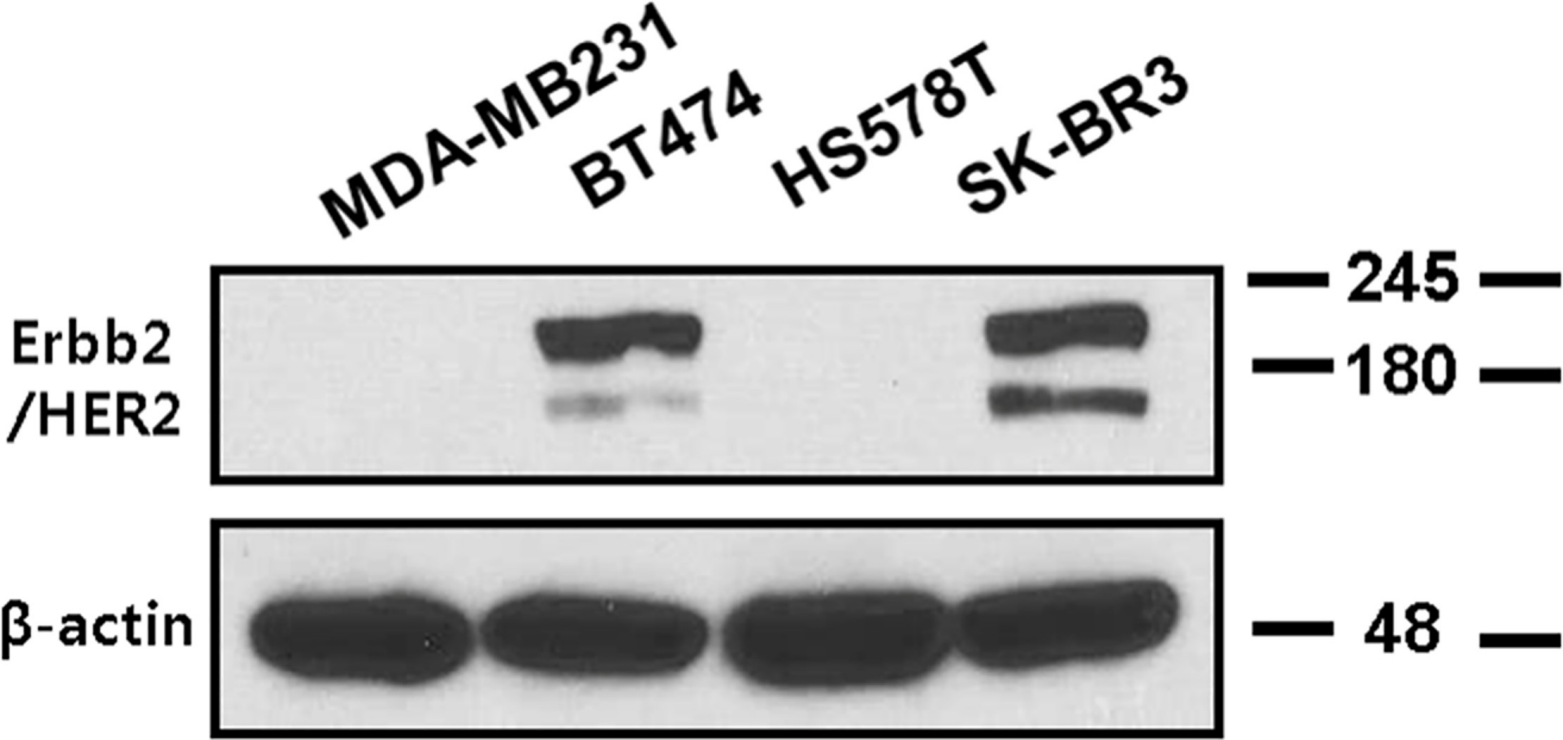
HER2 expression characteristics as determined by western blotting of human breast cancer cell lines. The BT474 and SK-BR3 cell lines highly expressed HER2, whereas MDA-MB231 and HS578T cells had no detectable HER2 expression. Beta-actin was used as a loading control.

Western blot and flow cytometry assays were performed to investigate the expression of HER2 in BT474 breast cancer cells. Western blot analysis confirmed overexpression of HER2 in BT474 and SKBR3 cells, which are known to overexpress HER2 due to gene amplification [33]. The negative control cell lines, MDA-MB231 and HS578T, showed no signal in the corresponding location (Fig 2).

**Fig 3:**
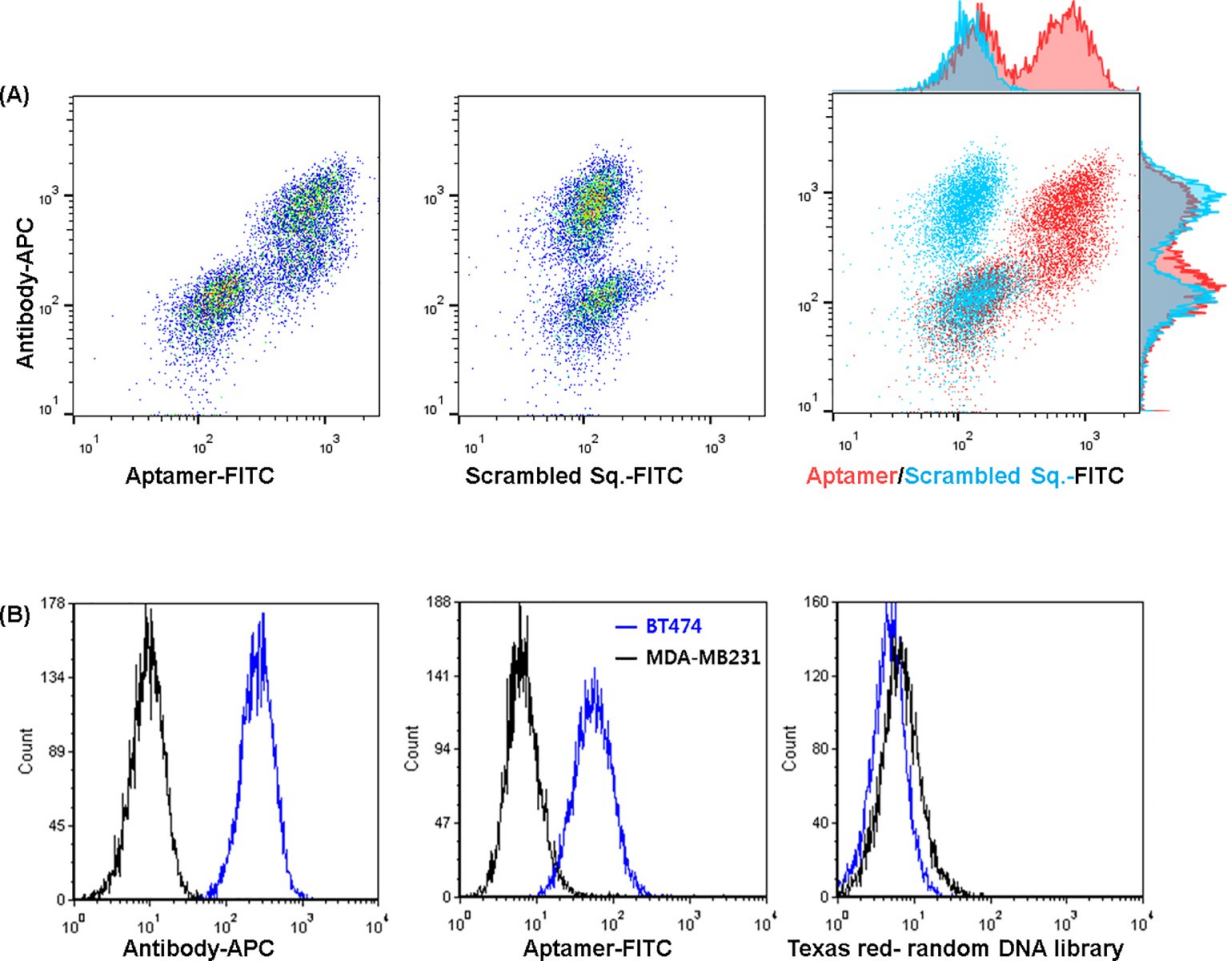
Flow cytometry analysis of breast cancer cell lines using HER2 antibody and aptamer. (A) Dot plots representing the fluorescence signals for BT474 (HER2-positive cells), MDA-MB231 (HER2-negative cells) from monoclonal antibody, and SH-1194-35 aptamer (red) or scrambled random sequence (blue). (B) Flow cytometric histogram of two cells using antibody, aptamer, and negative control.

As seen in Fig 3, HER2 antibody showed highly specific binding to HER2-positive BT474 cancer cells in flow cytometry analysis. Compared to antibody, HER2 aptamer (SH-1194-35) also had relatively strong binding to BT474 cells, while binding to MDA-MB231 cells was quite low. In addition, a random DNA oligonucleotide showed no significant binding preference for either cell line. These results suggested that the HER2 aptamer preferentially binds to HER2-positive breast cancer cells, possibly by recognizing the HER2 structure on the surface of these cells.

### Confocal microscopic analysis

**Fig 4:**
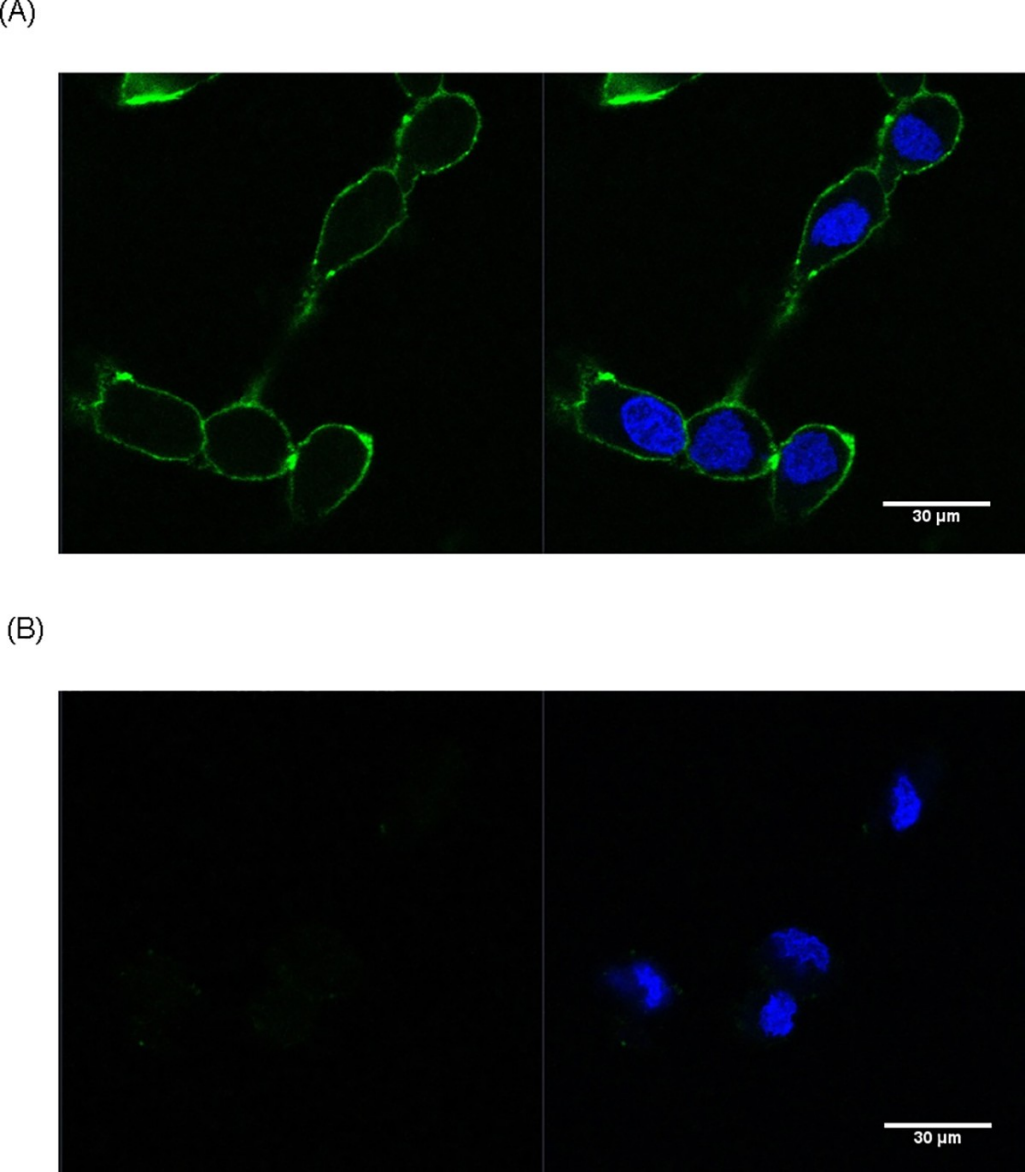
Confocal microscopic images of selected aptamer binding to HER2-positive cells. (A) BT474, HER2-positive breast cancer cells were incubated with FITC-labeled aptamer. (B) MDA-MB231 cancer cells were treated with the same aptamer. (Labeling, blue: DAPI; green: FITC-aptamer).

The binding of the selected aptamer to cells was further assessed by confocal microscopy (Fig 4). BT474 HER2-positive breast cancer cells were treated with conjugated aptamer. FITC-labeled SH-1194-35 aptamer was readily visualized on the cell surface, indicating occupation of the HER2 structure on the surface of these cells. The presence of the HER2 aptamer (green) was visualized along the cell membrane. In contrast, MDA-MB231 cells, the negative control, showed no significant fluorescence of the aptamer, indicating absence of HER2 expression. The SH-1194-35 aptamer was found to be able to bind HER2-positive breast cancer cells, with minimal binding to HER2-negative cells. The HER2 expression could be visualized by fluorescence-labeled aptamer.

### *In vivo* PET imaging, biodistribution, and immunohistochemistry

**Fig 5:**
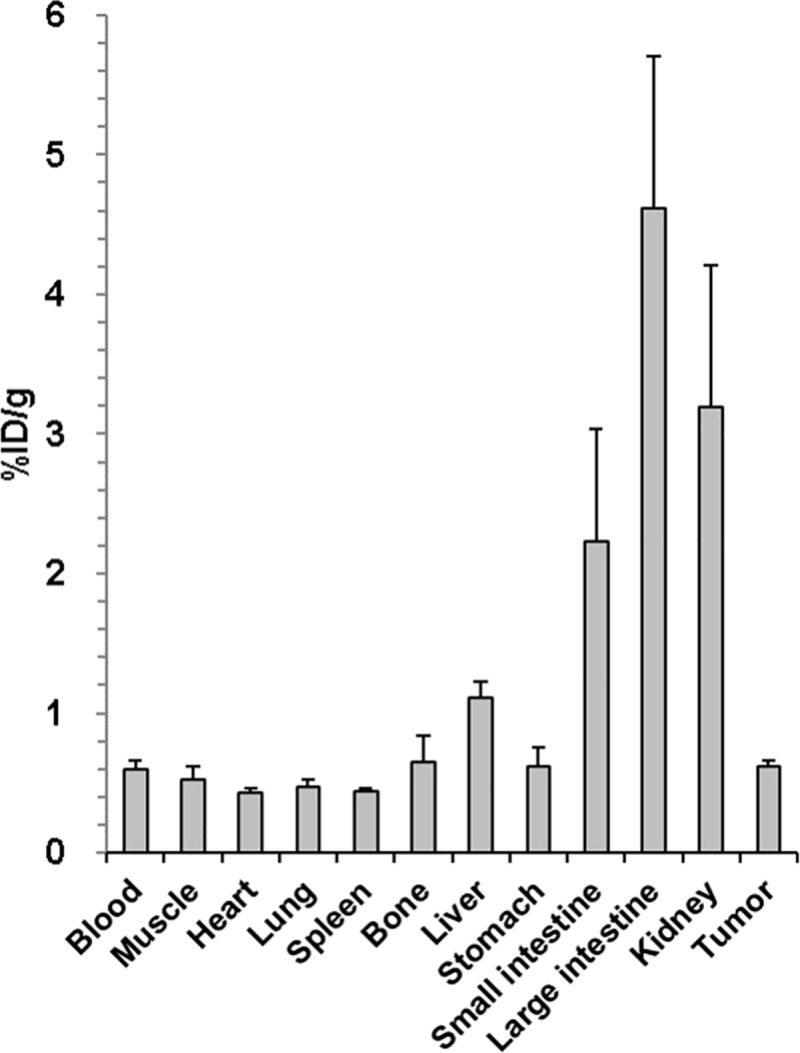
Biodistribution of ^18^F-labeled HER2 aptamer. Biodistribution study of ^18^F-labeled HER2 aptamer in BT474 tumor-bearing mice. Data are expressed as a percentage of injected activity per gram of tissue (%ID/g). Error bars, SD (N = 4).

Biodistribution was evaluated in tumor-bearing mice at 1-hour after injection of the ^18^F-labeled HER2-specific aptamer. After sacrificing the animal, radioactivity of each organ including tumor was measured using gamma counter and expressed as %ID/g (Fig 5). Tumor uptake of the ^18^F-labeled HER2-specific aptamer was 0.62±0.04 at 1 hour. Biodistribution study showed that the two major excretory systems of ^18^F-labeled HER2-specific aptamer are the kidneys and intestine.

**Fig 6:**
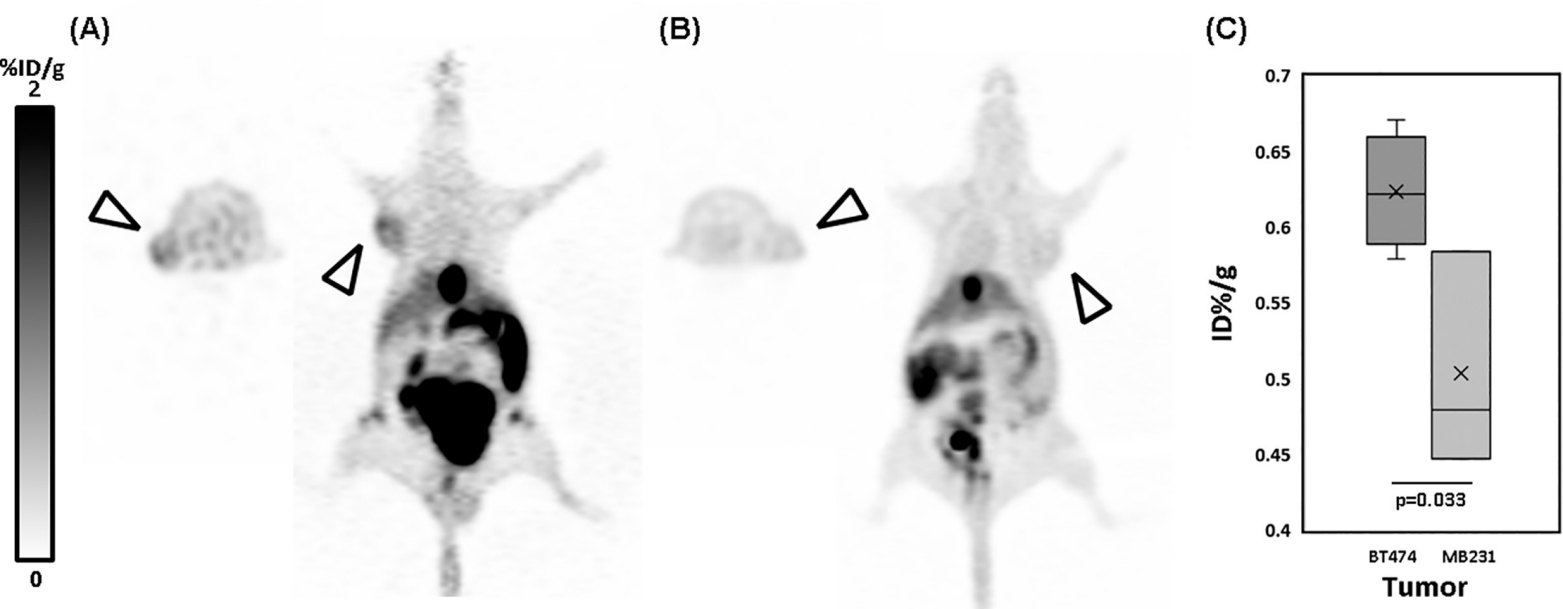
Representative images of *in vivo* ^18^F-labeled HER2-aptamer PET in HER2-positive and negative tumor-bearing mice. (A) HER2 overexpressing BT474 tumor shows increased uptake, compared to the (B) HER2-negative MB-MDA231 tumor. (C) %ID/g of tumor calculated from ^18^F-labeled HER2 aptamer. The %ID/g of aptamer in the BT474 tumor were significantly higher than those in the MDA-MD231 tumor.

Using animal micro PET, *in vivo* molecular images of BT474 tumor-bearing mice were taken at several time points. As seen in Fig 6, ^18^F-labeled HER2-specific aptamer PET showed significantly increased uptake into the tumor. In images taken at 120 minutes, the tumor was clearly labeled by SH-1194-35 aptamer in axial and coronal images (Fig 6A). Physiologic signal of radioactive tracer into the bowel and bladder was predominant, refiecting the two major clearance pathways of radiopharmaceuticals.

Fig 6 shows representative images of ^18^F-labeled HER2 aptamer PET in HER2-positive and negative tumor-bearing mice. BT474 tumors overexpressing HER2 showed higher radioisotope uptake than HER2-negative MB-MDA231 tumors. For semi-quantitative analysis, total activity of (nCi) each voxel- or volume-of-interest (VOI) was calculated. Comparison of tumor uptake between BT474 and MDA-MB231 cells demonstrated significantly in HER2 overexpressing BT474 tumors (Fig 6).

**Fig 7:**
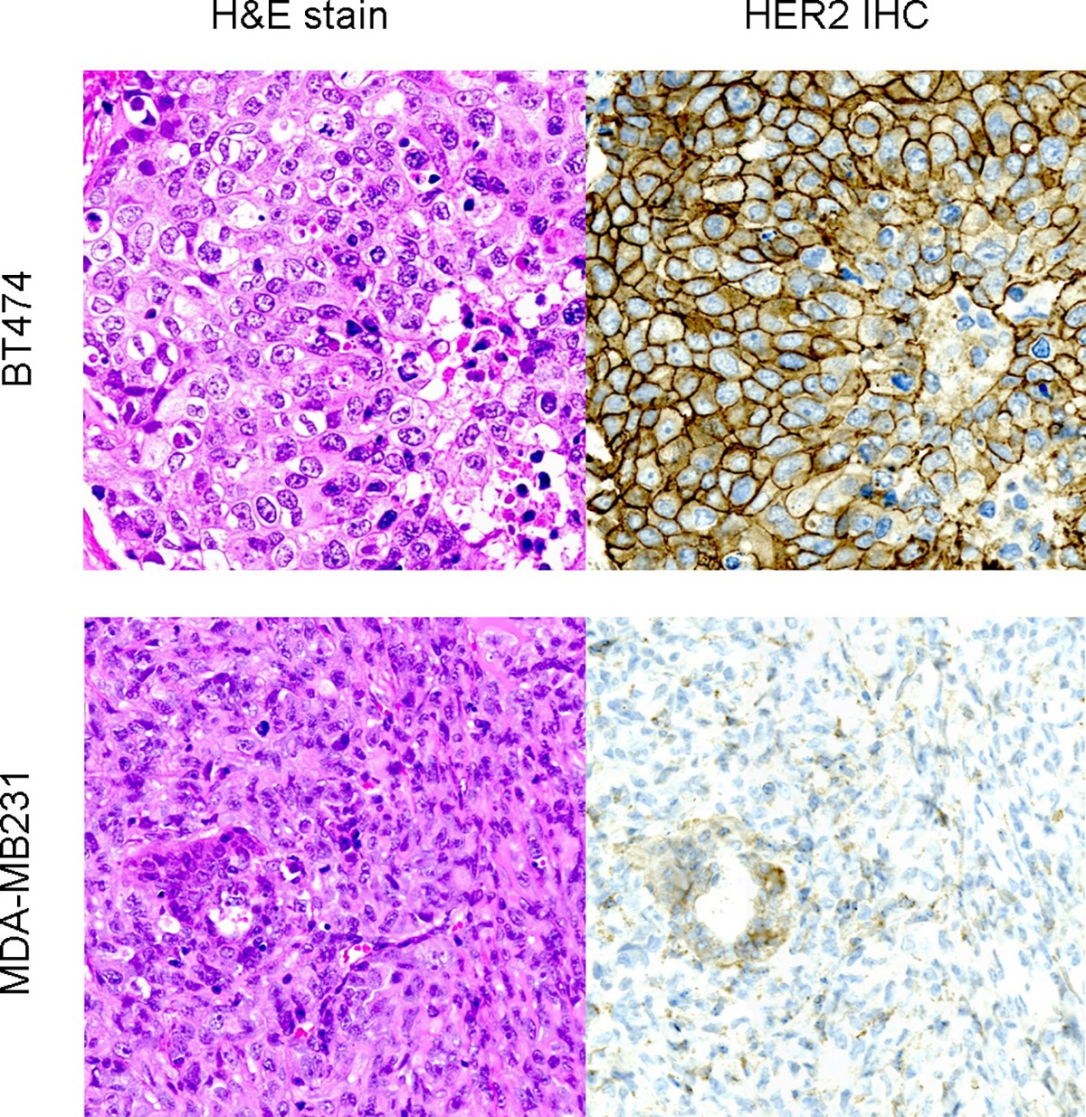
Representative staining results from H&E and IHC for HER2 (original magnification 400X). Immunohistochemically, the BT474 tumor cells (upper row) show strong membranous staining for HER2, compared to MDA-MB231 cells (lower row).

Immunohistochemistry confirmed the high expression of HER2 in BT474 tumors and low expression of HER2 in MDA-MB231 tumors extracted from each mouse group (Fig 7).

## Discussion

The primary objective of this study was to investigate tumor-specific PET imaging using radiolabeled aptamers. In this study, SH-1194-35, a HER2-targeted DNA aptamer, was successfully PET imaged *in vivo*. Since its first description in 1990 [1], numerous SELEX methods have been investigated and many novel aptamers have been developed. Among them, we chose HER2-targeted DNA aptamers as candidates for imaging agents. Previous studies have demonstrated the utility of HER2 aptamers in inhibiting tumorigenic growth, both by themselves [19] and by delivering cytotoxic drugs [18]. There are a few reports on the use of aptamers to image HER2-targeted cancer. Recently, an attempt to apply molecular imaging using a ^99m^Tc-labeled aptamer was reported, describing biodistribution data without images [34]. To our knowledge, the present study is the first report of HER2-targeted ^18^F-labeled PET imaging using specific aptamer. PET images of BT474 tumor-bearing mice showed reliable tumor-to-background ratios, which implies that the aptamer recognized the HER2 target *in vivo*.

In this study, flow cytometry and confocal microscopic analysis were performed to verify target-binding affinity of aptamers. There are many aptamers available for any given specific target. To determine which aptamer is appropriate for any particular study, assessment of affinity is required. SH-1194-35, a modified DNA aptamer designed to target HER2, showed superior performance in *in vitro* experiments, and thus, we adopted this aptamer for PET imaging.

Many therapeutic aptamers are being established in clinical trials for pharmaceutical safety and efficacy [35–38]. Among others, AS1411, a DNA aptamer targeting nucleolin, shows anti-tumor efficacy against renal cell carcinoma, and has completed a phase 2 trial [39]. Other aptamers, like ARC1779, targeting activated von Willebrand Factor (vWF), have verified efficacy against purpura and thrombotic thrombocytopenic disease. In addition, several RNA aptamer conjugates targeting PSMA [40] or designed to facilitate PSMA-targeted drug delivery [41] have reinvigorated research in the field. Likewise, due to tremendous translational potential for aptamers in clinical applications, there are many aptamers and aptamer-based drugs being evaluated for the treatment of a variety of disorders, involving those of coagulation, cancer, and inflammation.

Modification of magnetic nanoparticles or fluorescent agents with aptamers might provide good contrast agents for targeted fluorescence imaging and magnetic resonance imaging (MRI). Several *in vivo* MRI studies on tumor-bearing mice demonstrated efficient targeting of tumors [42, 43]. Since metabolic changes commonly occur before anatomical changes, PET has a clear diagnostic advantage over conventional anatomical techniques. In addition to its clinical utility, PET has a wide range of applications in basic research and preclinical arenas. For example, PET can be used to evaluate novel radiopharmaceuticals, effectiveness of new therapies, and biodistribution of pharmaceuticals. PET has key strengths in its depth of penetration, excellent sensitivity, quantitative data, and translatability from the pre-clinical to clinical stage [22]. However, there have been only a few studies that have attempted to establish PET imaging of aptamers. Recently, PET imaging of tenascin-C with a radiolabeled single-stranded DNA aptamer was reported [24]. A tenascin-C aptamer, labeled with ^18^F or ^64^Cu, demonstrated reliable tumor uptake, compared to a nonspecific scrambled aptamer. However, comparison with a conventional monoclonal antibody was not undertaken in that study.

The application of aptamers in cancer therapy is emerging and is being extensively studied at present. Our data suggest its potential for use as a target-specific molecular imaging tool for determining course of treatment. Aptamers or aptamer-based drugs may provide alternative options for the treatment of HER2-positive refractory malignancy, since aptamers may use different mechanisms than monoclonal antibodies do to occupy certain target molecules and may avoid provoking an immune reaction. In addition, because of their highly specific target affinity and applicability, aptamers might be ideal carriers for drugs or toxins. This has been demonstrated by Zhe Liu et al [18], who showed that a complex of aptamer and doxorubicin could selectively deliver doxorubicin to HER2-positive breast cancer cells, with minimal binding to HER2-negative cells.

There are several limitations inherent in this study. We have not yet determined the binding mechanism of the HER2 aptamer. Further research is needed to establish the molecular biologic mechanisms of aptamer. Additional methods may be implemented for PET image improvement in further studies. Although In vivo PET molecular imaging of BT474 tumor-bearing mice revealed significant higher uptake of the ^18^F-labeled HER2 specific aptamer into the tumor compared to the that of HER2-negative cell tumor (p = 0.033), physiologic bowel uptake is still predominant in whole body image. In order to eliminate physiologic uptake, a delayed image with clinically sufficient biologic half-life of a pharmaceutical is mandatory. One solution may be to perform chemical modification, including adding polyethylene glycol polymer chains (PEGylation), to aptamer to increase their stability. By attaching stabilizing material, it will be possible to improve the tumor/background ratio and reduce the bowel uptake in the delayed image. Also, further research is needed through complementary blocking and competition experiments. Meanwhile, the F-18 is a PET isotope with many advantages, but it is produced by cyclotron. Therefore, it is difficult to use in a place where the accessibility of the cyclotron is poor or the synthesis is not supported.

^18^F-labeled aptamer facilitated visualization of HER2-expression by *in vivo* molecular imaging. The PET images using ^18^F-labeled HER2 aptamer successfully showed target specific radioisotope uptake in the HER2 overexpressing tumor. These results suggest that radiolabeled HER2 aptamer may have potential applications in determining treatment strategies or in applying targeted therapy against HER2-positive breast cancer cells.

## References

[1] Tuerk C, Gold L. Systematic evolution of ligands by exponential enrichment: RNA ligands to bacteriophage T4 DNA polymerase. Science. 1990; 249: 505–10. 2200121 10.1126/science.2200121

[2] Ellington AD, Szostak JW. In vitro selection of RNA molecules that bind specific ligands. Nature. 1990; 346: 818–22. 10.1038/346818a0 1697402

[3] Stoltenburg R, Reinemann C, Strehlitz B. SELEX—a (r)evolutionary method to generate high-affinity nucleic acid ligands. Biomol Eng. 2007; 24: 381–403. 10.1016/j.bioeng.2007.06.001 17627883

[4] Spiridonova VA, Kopylov AM. DNA aptamers as radically new recognition elements for biosensors. Biochemistry (Mosc). 2002; 67: 706–9. 12126480 10.1023/a:1016110724564

[5] Jayasena SD. Aptamers: an emerging class of molecules that rival antibodies in diagnostics. Clin Chem. 1999; 45: 1628–50. 10471678

[6] Bock LC, Griffin LC, Latham JA, Vermaas EH, Toole JJ. Selection of single-stranded DNA molecules that bind and inhibit human thrombin. Nature. 1992; 355: 564–6. 10.1038/355564a0 1741036

[7] Bates PJ, Laber DA, Miller DM, Thomas SD, Trent JO. Discovery and development of the G-rich oligonucleotide AS1411 as a novel treatment for cancer. Exp Mol Pathol. 2009; 86: 151–64. 10.1016/j.yexmp.2009.01.004 19454272 PMC2716701

[8] Lupold SE, Hicke BJ, Lin Y, Coffey DS. Identification and characterization of nuclease-stabilized RNA molecules that bind human prostate cancer cells via the prostate-specific membrane antigen. Cancer Res. 2002; 62: 4029–33. 12124337

[9] Daniels DA, Chen H, Hicke BJ, Swiderek KM, Gold L. A tenascin-C aptamer identified by tumor cell SELEX: systematic evolution of ligands by exponential enrichment. Proc Natl Acad Sci U S A. 2003; 100: 15416–21. 10.1073/pnas.2136683100 14676325 PMC307582

[10] James W. Aptamers in the virologists' toolkit. J Gen Virol. 2007; 88: 351–64. 10.1099/vir.0.82442-0 17251551

[11] Doggrell SA. Pegaptanib: the first antiangiogenic agent approved for neovascular macular degeneration. Expert Opin Pharmacother. 2005; 6: 1421–3. 10.1517/14656566.6.8.1421 16013991

[12] Dausse E, Da Rocha Gomes S, Toulme JJ. Aptamers: a new class of oligonucleotides in the drug discovery pipeline? Curr Opin Pharmacol. 2009; 9: 602–7. 10.1016/j.coph.2009.07.006 19717337

[13] Mitri Z, Constantine T, O'Regan R. The HER2 Receptor in Breast Cancer: Pathophysiology, Clinical Use, and New Advances in Therapy. Chemother Res Pract. 2012; 2012: 743193 10.1155/2012/743193 23320171 PMC3539433

[14] Burstein HJ. The distinctive nature of HER2-positive breast cancers. N Engl J Med. 2005; 353: 1652–4. 10.1056/NEJMp058197 16236735

[15] Tan M, Yu D. Molecular mechanisms of erbB2-mediated breast cancer chemoresistance. Adv Exp Med Biol. 2007; 608: 119–29. 17993237 10.1007/978-0-387-74039-3_9

[16] Baselga J, Swain SM. Novel anticancer targets: revisiting ERBB2 and discovering ERBB3. Nat Rev Cancer. 2009; 9: 463–75. 10.1038/nrc2656 19536107

[17] Dastjerdi K, Tabar GH, Dehghani H, Haghparast A. Generation of an enriched pool of DNA aptamers for an HER2-overexpressing cell line selected by Cell SELEX. Biotechnol Appl Biochem. 2011; 58: 226–30. 10.1002/bab.36 21838796

[18] Liu Z, Duan JH, Song YM, Ma J, Wang FD, Lu X, Yang XD. Novel HER2 aptamer selectively delivers cytotoxic drug to HER2-positive breast cancer cells in vitro. J Transl Med. 2012; 10: 148 10.1186/1479-5876-10-148 22817844 PMC3583217

[19] Mahlknecht G, Maron R, Mancini M, Schechter B, Sela M, Yarden Y. Aptamer to ErbB-2/HER2 enhances degradation of the target and inhibits tumorigenic growth. Proc Natl Acad Sci U S A. 2013; 110: 8170–5. 10.1073/pnas.1302594110 23630281 PMC3657787

[20] Mahlknecht G, Sela M, Yarden Y. Aptamer Targeting the ERBB2 Receptor Tyrosine Kinase for Applications in Tumor Therapy. Methods Mol Biol. 2015; 1317: 3–15. 10.1007/978-1-4939-2727-2_1 26072398

[21] Moosavian SA, Jaafari MR, Taghdisi SM, Mosaffa F, Badiee A, Abnous K. Development of RNA aptamers as molecular probes for HER2(+) breast cancer study using cell-SELEX. Iran J Basic Med Sci. 2015; 18: 576–86. 26221481 PMC4509953

[22] James ML, Gambhir SS. A molecular imaging primer: modalities, imaging agents, and applications. Physiol Rev. 2012; 92: 897–965. 10.1152/physrev.00049.2010 22535898

[23] Hicke BJ, Stephens AW, Gould T, Chang YF, Lynott CK, Heil J, Borkowski S, Hilger CS, Cook G, Warren S, Schmidt PG. Tumor targeting by an aptamer. J Nucl Med. 2006; 47: 668–78. 16595502

[24] Jacobson O, Yan X, Niu G, Weiss ID, Ma Y, Szajek LP, Shen B, Kiesewetter DO, Chen X. PET imaging of tenascin-C with a radiolabeled single-stranded DNA aptamer. J Nucl Med. 2015; 56: 616–21. 10.2967/jnumed.114.149484 25698784 PMC5226408

[25] Pala K, Serwotka A, Jelen F, Jakimowicz P, Otlewski J. Tumor-specific hyperthermia with aptamer-tagged superparamagnetic nanoparticles. Int J Nanomedicine. 2014; 9: 67–76. 10.2147/ijn.s52539 24379664 PMC3872225

[26] Zuker M. Mfold web server for nucleic acid folding and hybridization prediction. Nucleic Acids Res. 2003; 31: 3406–15. 12824337 10.1093/nar/gkg595 PMC169194

[27] Kibbe WA. OligoCalc: an online oligonucleotide properties calculator. Nucleic Acids Res. 2007; 35: W43–6. 10.1093/nar/gkm234 17452344 PMC1933198

[28] Tang G, Zeng W, Yu M, Kabalka G. Facile synthesis of N-succinimidyl 4-[18F]fluorobenzoate ([18F]SFB) for protein labeling. Journal of Labelled Compounds and Radiopharmaceuticals. 2008; 51: 68–71. 10.1002/jlcr.1481

[29] Tang G, Tang X, Wang X. A facile automated synthesis of N-succinimidyl 4-[18F]fluorobenzoate ([18F]SFB) for 18F-labeled cell-penetrating peptide as PET tracer. Journal of Labelled Compounds and Radiopharmaceuticals. 2010; 53: 543–7. 10.1002/jlcr.1758

[30] Scott PJH, Shao X. Fully automated, high yielding production of N-succinimidyl 4-[18F]fluorobenzoate ([18F]SFB), and its use in microwave-enhanced radiochemical coupling reactions. Journal of Labelled Compounds and Radiopharmaceuticals. 2010; 53: 586–91. 10.1002/jlcr.1785

[31] Flagothier J, Kaisin G, Mercier F, Thonon D, Teller N, Wouters J, Luxen A. Synthesis of two new alkyne-bearing linkers used for the preparation of siRNA for labeling by click chemistry with fluorine-18. Appl Radiat Isot. 2012; 70: 1549–57. 10.1016/j.apradiso.2012.04.022 22732389

[32] Ramenda T, Steinbach J, Wuest F. 4-[18F]Fluoro-N-methyl-N-(propyl-2-yn-1-yl)benzenesulfonamide ([18F]F-SA): a versatile building block for labeling of peptides, proteins and oligonucleotides with fluorine-18 via Cu(I)-mediated click chemistry. Amino Acids. 2013; 44: 1167–80. 10.1007/s00726-012-1450-4 23306450

[33] Kraus MH, Popescu NC, Amsbaugh SC, King CR. Overexpression of the EGF receptor-related proto-oncogene erbB-2 in human mammary tumor cell lines by different molecular mechanisms. Embo j. 1987; 6: 605–10. 3034598 10.1002/j.1460-2075.1987.tb04797.x PMC553440

[34] Varmira K, Hosseinimehr SJ, Noaparast Z, Abedi SM. An improved radiolabelled RNA aptamer molecule for HER2 imaging in cancers. J Drug Target. 2014; 22: 116–22. 10.3109/1061186X.2013.839688 24098950

[35] Bouchard PR, Hutabarat RM, Thompson KM. Discovery and development of therapeutic aptamers. Annu Rev Pharmacol Toxicol. 2010; 50: 237–57. 10.1146/annurev.pharmtox.010909.105547 20055704

[36] Sun H, Zhu X, Lu PY, Rosato RR, Tan W, Zu Y. Oligonucleotide aptamers: new tools for targeted cancer therapy. Mol Ther Nucleic Acids. 2014; 3: e182 10.1038/mtna.2014.32 25093706 PMC4221593

[37] Lao YH, Phua KK, Leong KW. Aptamer nanomedicine for cancer therapeutics: barriers and potential for translation. ACS Nano. 2015; 9: 2235–54. 10.1021/nn507494p 25731717

[38] Sun H, Zu Y. A Highlight of Recent Advances in Aptamer Technology and Its Application. Molecules. 2015; 20: 11959–80. 10.3390/molecules200711959 26133761 PMC6331864

[39] Rosenberg JE, Bambury RM, Van Allen EM, Drabkin HA, Lara PN Jr., Harzstark AL, Wagle N, Figlin RA, Smith GW, Garraway LA, Choueiri T, Erlandsson F, Laber DA. A phase II trial of AS1411 (a novel nucleolin-targeted DNA aptamer) in metastatic renal cell carcinoma. Invest New Drugs. 2014; 32: 178–87. 10.1007/s10637-013-0045-6 24242861 PMC4560460

[40] Dassie JP, Hernandez LI, Thomas GS, Long ME, Rockey WM, Howell CA, Chen Y, Hernandez FJ, Liu XY, Wilson ME, Allen LA, Vaena DA, Meyerholz DK, et al Targeted inhibition of prostate cancer metastases with an RNA aptamer to prostate-specific membrane antigen. Mol Ther. 2014; 22: 1910–22. 10.1038/mt.2014.117 24954476 PMC4429728

[41] Xu W, Siddiqui IA, Nihal M, Pilla S, Rosenthal K, Mukhtar H, Gong S. Aptamer-conjugated and doxorubicin-loaded unimolecular micelles for targeted therapy of prostate cancer. Biomaterials. 2013; 34: 5244–53. 10.1016/j.biomaterials.2013.03.006 23582862 PMC3960945

[42] Lim EK, Kim B, Choi Y, Ro Y, Cho EJ, Lee JH, Ryu SH, Suh JS, Haam S, Huh YM. Aptamer-conjugated magnetic nanoparticles enable efficient targeted detection of integrin alphavbeta3 via magnetic resonance imaging. J Biomed Mater Res A. 2014; 102: 49–59. 10.1002/jbm.a.34678 23568770

[43] Hu H, Dai A, Sun J, Li X, Gao F, Wu L, Fang Y, Yang H, An L, Wu H, Yang S. Aptamer-conjugated Mn3O4@SiO2 core-shell nanoprobes for targeted magnetic resonance imaging. Nanoscale. 2013; 5: 10447–54. 10.1039/c3nr03490a 24057072

